# fMRI Neurofeedback-Enhanced Cognitive Reappraisal Training in Depression: A Double-Blind Comparison of Left and Right vlPFC Regulation

**DOI:** 10.3389/fpsyt.2021.715898

**Published:** 2021-08-23

**Authors:** Micha Keller, Jana Zweerings, Martin Klasen, Mikhail Zvyagintsev, Jorge Iglesias, Raul Mendoza Quiñones, Klaus Mathiak

**Affiliations:** ^1^Department of Psychiatry, Psychotherapy and Psychosomatics, School of Medicine, RWTH Aachen University, Aachen, Germany; ^2^Interdisciplinary Training Centre for Medical Education and Patient Safety—AIXTRA, Medical Faculty, RWTH Aachen University, Aachen, Germany; ^3^Department of Cognitive Neuroscience, Cuban Center for Neuroscience, Havana, Cuba; ^4^JARA-Brain, Research Center Jülich, Jülich, Germany

**Keywords:** real-time fMRI neurofeedback, depression, emotion regulation, cognitive reappraisal, lateral PFC

## Abstract

Affective disorders are associated with maladaptive emotion regulation strategies. In particular, the left more than the right ventrolateral prefrontal cortex (vlPFC) may insufficiently regulate emotion processing, e.g., in the amygdala. A double-blind cross-over study investigated NF-supported cognitive reappraisal training in major depression (*n* = 42) and age- and gender-matched controls (*n* = 39). In a randomized order, participants trained to upregulate either the left or the right vlPFC during cognitive reappraisal of negative images on two separate days. We wanted to confirm regional specific NF effects with improved learning for left compared to right vlPFC (ClinicalTrials.gov NCT03183947). Brain responses and connectivity were studied with respect to training progress, gender, and clinical outcomes in a 4-week follow-up. Increase of vlPFC activity was stronger after NF training from the left- than the right-hemispheric ROI. This regional-specific NF effect during cognitive reappraisal was present across patients with depression and controls and supports a central role of the left vlPFC for cognitive reappraisal. Further, the activity in the left target region was associated with increased use of cognitive reappraisal strategies (*r* = 0.48). In the 4-week follow-up, 75% of patients with depression reported a successful application of learned strategies in everyday life and 55% a clinically meaningful symptom improvement suggesting clinical usability.

## Introduction

In everyday life we are frequently challenged by situations that evoke negative emotions. As part of an adaptive response to these encounters we may change the experience and expression of our emotions by using emotion regulation (1). However, the success of our emotional response modulation may depend on our mental condition. For instance, emotion dysregulation is a characteristic symptom of major depressive disorder (MDD). Increased selective attention and processing of negative mood-congruent stimuli as well as maladaptive emotional responses may propel the development and recurrence of depressive episodes (2–4). A recent meta-analysis indicates that patients with depression show abnormal recruitment of the emotion regulation brain network during cognitive reappraisal of negative images (5). Furthermore, patients with depression often find it difficult to spontaneously utilize these strategies in everyday situations (6, 7) and methods are needed to bridge the gap between theory and everyday life application. A recent study by (8) has shown that even a single session of real-time functional magnetic imaging neurofeedback (rtfMRI-NF) could enhance the transfer of skills learned by patients with depression during CBT to real-world situations. RtfMRI-NF is a novel technique by which individuals with psychiatric disorders can learn to voluntarily self-regulate their brain signal in areas amongst others involved in the neural circuitry of emotion regulation and thereby induce changes in neural plasticity (9–13). Therefore, providing NF can inform neuroscience-based interventions for emotion dysregulation and may offer a more specific clinical tool for augmenting self-regulation in patients with depression by strengthening the monitoring within the emotion regulation process (7, 14).

Cognitive reappraisal is essential for psychological functioning and well-being and has been linked to lower levels of psychopathology (15). This strategy is focused on lowering the valence of negative situations by reinterpreting its meaning in a more positive way (1). This process is associated with a decrease in negative and an increase in positive affect (16). It mediates the relationship between stress and depressive symptoms (17) and may be beneficial on the long run by decreasing the impact of recurrent negative stimuli (18). Yet, patients with depression tend to overuse maladaptive emotion regulation strategies such as rumination as well as suppression of emotional experiences (19–21) which adds to the negativity bias of attention, information processing and memory formation (22–24). Emotion dysregulation may facilitate the development and recurrence of symptoms of depression (25, 26). As cognitive reappraisal strategies are not frequently used by patients with depression (20, 27–29), training reappraisal ability is an established component of cognitive behavioral therapy (CBT) (30, 31). CBT modulates the neural circuitry of emotions (32). Furthermore, self-reported reappraisal success following CBT in patients with social anxiety disorder predicted symptom reductions (33). Despite of promising effects, patients often struggle to transfer these cognitive strategies from theoretical to real-world applications (34). This can be supported by web-based interventions that train cognitive reappraisal on a regular basis by receiving feedback from peers (35) or instead by rtfMRI NF training (8).

On the neurobiological level, the downregulation of emotional reactivity in healthy participants is associated with a widespread network including frontal, parietal as well as subcortical regions (36–38). In this network, the vlPFC seems to play a pivotal role for the process of emotion regulation due to its dense structural and functional connections to other prefrontal, somatosensory, motor and language areas and its link to response selection and inhibition (37). Furthermore, gray matter volume reductions in the vlPFC have been found in patients with depression (39). Especially reinterpretation (compared to distancing) of negative affective content has been related to peak activation in the left vlPFC and the left STG (40). The vlPFC relays the need to regulate to a fronto-parietal cognitive control network (dlPFC, pre-SMA, STG, posterior parietal cortex) which is subsequently involved in the execution of regulation (37). Cognitive reappraisal or related emotion regulation strategies that are applied in response to emotional provocation, modulate the semantic representation of an emotional stimulus and the emotional responding through subcortical pathways (36, 41). Interestingly, gender differences in cognitive regulation such as more rumination and catastrophizing in females (42) and alcohol for coping in men (43) have also been observed on the neural level (44). Meta-analyses have investigated differences during emotion regulation between healthy controls and individuals with depression (5, 40). Hyperactivity in the amygdala during downregulation of negative stimuli has consistently been reported to be specific for affective disorders which indicates increased bottom-up responding or ineffective modulatory capacity of regulatory networks during emotion appraisals (5). Furthermore, the vlPFC and dlPFC (5) as well as the left STG (40) show less activation in patients with mood disorders which may be related to a dysfunctional management of attentional and inhibitory resources and make these areas potential targets for NF training.

Neuromodulation approaches of emotion regulation networks not only underline the causal role of the lPFC for cognitive reappraisal but also show promise for improving depressive symptomatology. For instance, repetitive transcranical magnetic stimulation (rTMS) over the lPFC is an evidence-based treatment for depression which is successfully applied in treatment refractory depression (45, 46). In a meta-analysis of therapeutic responses to brain stimulation in depression (47), excitatory rTMS seemed to perform better at the left than the right lPFC (odds ratio: 1.89) and the reversed pattern was observed after inhibitory stimulation (OR: 3.29). These comparisons, however, did not encompass direct comparison within studies and failed to show a statistically significant difference. The causal role of the vlPFC in specific is supported by experimental TMS studies that demonstrated a facilitation of reappraisal after vlPFC stimulation (48, 49). In a similar vein, transcranial direct current stimulation (tDCS) of the vlPFC may influence emotion regulation. For instance, excitatory tDCS stimulation of the vlPFC facilitated the downregulation of negative emotions using cognitive reappraisal compared to dlPFC or sham stimulation (50–52). Taken together, these findings further support a key role of the lPFC for mood disorders and show that especially the vlPFC is a suitable target for NF-supported emotion regulation training.

EEG and fMRI studies indicate that the laterality of neural activation may be indicative of affective style. According to the frontal asymmetry model, relatively higher right frontal alpha power (more left PFC neural activity) suggests approach-related emotions while higher left frontal alpha power (more right PFC neural activity) may relate to withdrawal-related emotions (53, 54). Furthermore, electrophysiological studies have suggested that left-sided frontal alpha asymmetry (FAA; higher alpha power in the left compared to right frontal channels) may be a biomarker of depression [e.g., (55)]. Even though the relation of FAA and depression may be only of small magnitude and use of FAA as a biomarker of depression seems too farfetched (56), frontal EEG asymmetry may be used as index of emotion regulation capability (57). For instance, a shift toward left asymmetry induced by mindfulness training was associated with improved responses during emotional challenges (58). In an EEG emotion regulation paradigm, individuals with higher capacity for reappraisal showed more left-lateralized (ventro) lateral PFC activation (59). Consequently, recruitment of left-lateralized PFC areas was associated with creating alternative appraisals of negative situations. Furthermore, in an fMRI investigation of laterality, less activation of the left relative to right IFG was associated with poor performance on an emotion perception task (60). Lastly, in a combined rtfMRI-EEG-NF paradigm, patients with depression achieved a shift toward left frontal activation as well as simultaneous changes in amygdala neural activity laterality, indicating an enhancement of approach motivation (61). These studies indicate that training left relative to right hemispheric IFG activity may be beneficial for patients with depression. Nevertheless, the right vlPFC seems to exhibit similar effects as the left hemispheric cognate in social perception (48, 50).

So far, several rtfMRI NF studies training self-regulation of emotion processing areas suggest this method may have an added benefit for the treatment of depression (62, 63). A reduction of depressive symptomatology has been achieved by training upregulation of the amygdala (64, 65) and of areas responsive to positive mental imagery (66, 67). To date, only one NF study trained patients with depression to downregulate brain activity in response to negative stimuli. Hamilton et al. (68) observed that providing patients with real [salience network (SN) node] compared to yoked feedback during an emotion regulation task led to decreased SN responses and greater reduction of emotional responses to negative stimuli. Successful downregulation of subcortical emotional processing areas such as the amygdala has also been shown in healthy individuals (69). Furthermore, limbic activity can indirectly be influenced by increasing top-down regulation. For instance, Sarkheil et al. (70) showed that attenuation of amygdala responses during emotion regulation training in healthy individuals could be enhanced by receiving feedback from the lateral PFC. In a similar NF-supported emotion regulation paradigm, Zweerings et al. (71) found reduced amygdala responses when receiving NF vs. not receiving NF in patients with posttraumatic stress disorder. Furthermore, the level of amygdala attenuation could be associated with improved symptomatology and negative affect 4 weeks later. In a preliminary report by Takamura et al. (72), rtfMRI NF of the left dlPFC was associated with clinical measures of depression. Patients with depression show deficient top-down regulation and may profit from NF-guided cognitive reappraisal training which may ease the transition from laboratory settings to the application in daily life. Emotion regulation strategies such as cognitive reappraisal are difficult to apply in emotionally demanding (high stress) situations and ways to train cognitive reappraisal more effectively may be beneficial. RtfMRI NF provides an objective neural indicator of regulation success that may improve identification of successful regulation strategies and strengthen experienced self-efficacy.

In the current study, we investigated the feasibility of a NF-guided cognitive reappraisal training in patients with depression using a double-blind cross-over design. On two separate NF training days, participants upregulated either the left or right vlPFC in response to negative pictures by applying strategies of cognitive reappraisal. Based on the reviewed fMRI and EEG literature, we hypothesized that the left compared to right vlPFC may be more important for the emotion regulation process. Therefore, the right vlPFC was chosen as active control condition as it is known to be involved in cognitive reappraisal, however, it is less consistently observed and activation levels tend to be lower (37, 70, 71). An active control condition can circumvent the problem of causing frustration as regulation is expected to be possible in both conditions. Accordingly, motivation levels between conditions are likely comparable. Following this line of interpretation, we hypothesized that (1) learning of regulation would be enhanced by feedback from the left compared to the right vlPFC. This hypothesis reflected the double-blind randomization condition and was registered a-priori as primary outcome (NCT03183947). To further explore effects of the training, we (2) investigated neurofeedback effects on the whole-brain level as well as (3) task-dependent changes in connectivity patterns. Lastly, we (4) hypothesized that successful regulation during NF would be accompanied by changes in measures of mood, emotion regulation and depressive symptomatology.

## Method

### Participants

Forty-two patients with major depressive disorder (MDD) and 39 age and gender matched healthy individuals completed the rt-fMRI NF training. All participants had adequate knowledge of the German language, normal or corrected to normal vision and were right-handed. Exclusion criteria were contraindications to MRI, traumatic brain injury, neurological illness, serious suicidal ideation, or inability for informed consent. Furthermore, healthy participants were not included if they had a history of psychiatric illness assessed with the screening questions of the German version of the Structured Clinical Interview for assessment of DSM-IV-TR criteria [SCID-I; (73)]. All patients fulfilled the formal criteria of a diagnosis of an acute MDD such as established by a psychiatrist. We included patients meeting criteria for comorbid disorders in addition to MDD. The diagnosis was confirmed according to the Diagnostic and Statistical Manual of Mental Disorders (DSM-IV-TR; American Psychiatric Association) (73) criteria by an experienced psychologist. Average number of MDEs was 4.5 (± 5.7) and average duration of illness was 8.4 years (± 8.0). Patients had a stable level of medication for at least 1 week prior to inclusion and during the time of the study. For the analysis, three patients were excluded due to remission at the time of NF training and two control participants were excluded due to excessive head movement during measurements and lost data (technical problems in real-time processing). Accordingly, the analyzed sample consisted of 39 patients (35.2 ± 2.2 years; 17 female) and 37 healthy controls (32.3 ± 2.1 years; 15 female). Groups did not differ with respect to age [*t*_(74)_ = −0.96, *p* = 0.34] and years of education [*t*_(74)_ = 0.78, *p* = 0.44] (Table 1 for more information). The study was pre-registered at clinicaltrials.gov (NCT7171). Due to constraints in recruitment procedures the subsample of patients with schizophrenia has not been completed until now. The study was approved by the local Ethics Committee of the RWTH Aachen (EK 050/17) and all participants provided written informed consent.

**Table 1:** Demographic and clinical data.

	**MDD** **(** ***n*** **=** **39)**		**HC** **(** ***n*** **=** **37)**			**Comparison**	
	**Mean**	**SD**	**Mean**	**SD**	***Df***	***t***	***p***
Age (years)	35.2	13.6	32.3	12.8	74	−0.96	0.34
Education (years)	14.3	2.5	14.8	2.6	74	0.78	0.44
Parental education (years)	13.7	2.7	13.6	3.2	67	−0.16	0.88
Socioeconomic status (monthly income in Euro)	1,372	1,191	1,115	1,034	72	−0.99	0.33
**Clinical characteristics**							
**ERQ** ^a^ **-**							
Reappraisal	3.8	1.3	5.0	0.8	71	4.7	**<0.001**
Suppression	4.3	1.3	3.6	1.1	71	−2.4	**<0.05**
**HFERST** ^b^ **-**							
Reappraisal	2.7	0.8	3.7	0.6	71	5.7	**<0.001**
Acceptance	2.8	1.1	3.8	0.7	71	5.0	**<0.001**
Problem solving	3.7	1.0	4.3	0.5	71	3.2	**<0.01**
Social support	2.4	1.2	3.4	1.0	71	3.8	**<0.001**
Rumination	4.0	0.7	3.1	0.7	71	−5.1	**<0.001**
Avoidance	3.5	1.2	2.8	0.8	71	−2.9	**<0.01**
Experience suppression	2.8	0.7	2.4	0.6	71	−2.7	**<0.05**
Expressive suppression	3.4	1.0	3.0	0.7	71	−1.8	0.08
Verbal IQ (WST^c^)	31.0	5.0	31.9	5.4	74	0.73	0.47
Digit span	15.2	4.3	15.2	3.8	74	0.04	0.97
Digit symbol test	55.3	13.9	58.1	11.4	72	0.96	0.34
Anhedonia (Chapman)	16.2	5.4	10.6	5.8	74	−4.3	**<0.001**
HADS^d^ - Anxiety	10.9	3.2	4.7	4.6	74	−6.9	**<0.001**
HADS - Depression	10.9	3.9	2.8	3.6	74	−9.5	**<0.001**
HAM-D^e^	16.5	7.5					
BDI-II^f^ baseline	26.8	11.5	3.8	4.2	73	−10.8	**<0.001**
Average number of MDEs	4.5	5.7					
Duration of illness	8.5	8.0					
Antidepressants^g^ (*N* = 35)	198.0	137.7					
Antipsychotics^g^ (*N* = 5)	48.0	68.9					
**Comorbidities** ***N*** **(%)**							
Dysthimia	1 (2.56)						
Anxiety Disorders	24 (61.5)						
Eating Disorders	2 (5.13)						

*ERQ^a^, emotion regulation questionnaire; HFERST^b^, heidelberg Form for emotion regulation strategies; WST^c^, wortschatztest; HADS^d^, hospital anxiety and depression scale; HAM-D^e^, hamilton depression rating scale; BDI-II^f^, beck depression inventory-II; ^g^, medication is reported as percentage of the defined daily dose. Bold values indicate significant difference between healthy individuals and patients with depression*.

### Experimental Procedure

**Figure 1:**
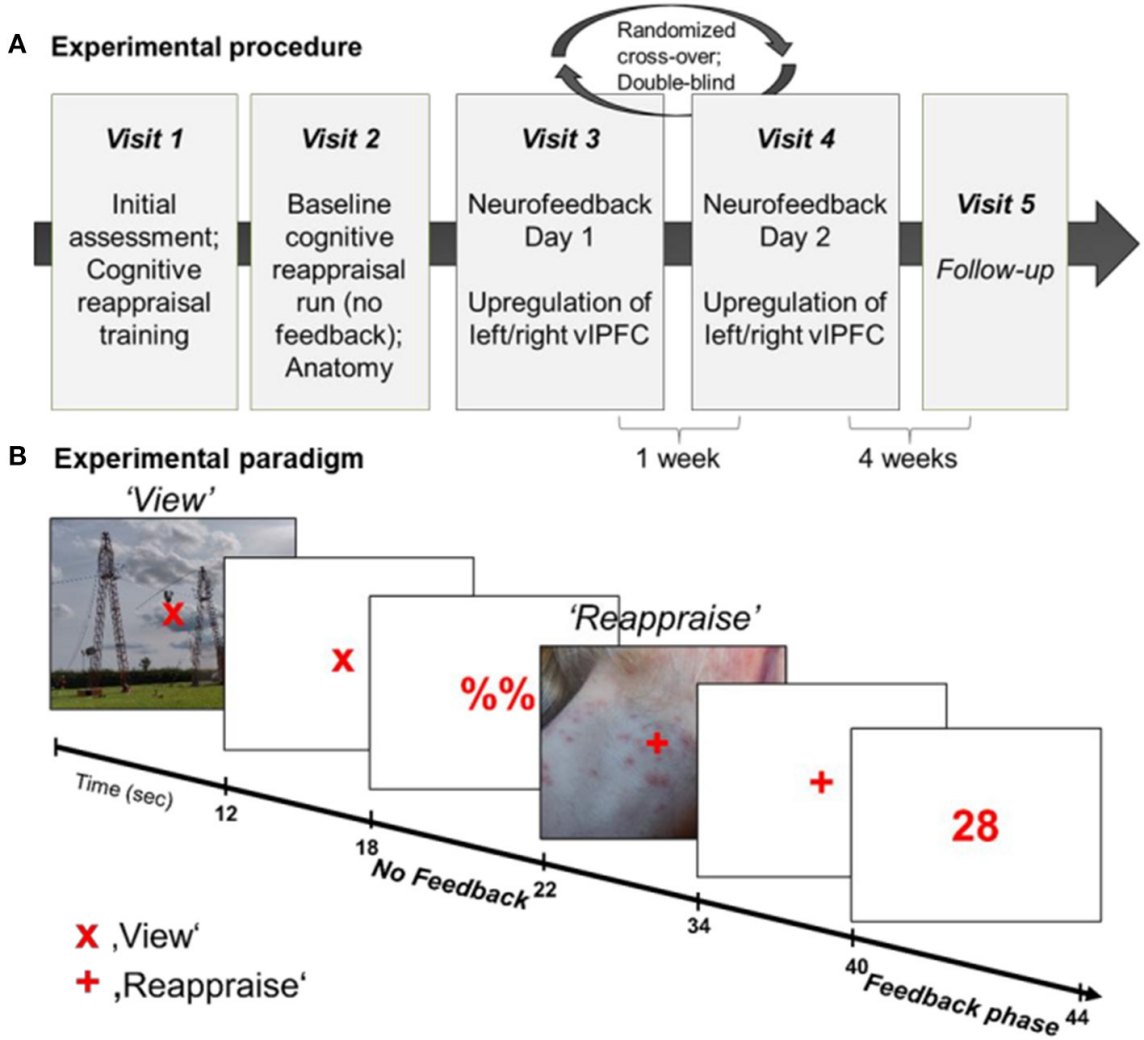
**(A)** Experimental procedure. The NF training entailed 5 visits to the lab, starting with initial assessment and cognitive reappraisal training (Visit 1), followed by baseline cognitive reappraisal runs and anatomical recordings (Visit 2), two NF training days (Visit 3 and 4) and a follow-up assessment (Visit 5). **(B)** Experimental paradigm during NF training. All participants completed 4 NF runs on each of the two NF days. Each NF run entailed 9 view-regulate cycles. During “view” trials (“x”), participants passively viewed the picture and could allow spontaneous thoughts and emotions. On “regulate” trials (“+”) participants reappraised the picture to reduce the negative affect and upregulate the BOLD signal in the target area. Each picture was presented for 12 s, followed by a 6-s fixation cross and 4-s (pseudo-) neurofeedback interval.

In a randomized, double-blind cross-over design (see Figure 1A), participants were trained to upregulate their brain activation in the anatomically defined region of interest (ROI) (left or right ventrolateral prefrontal cortex, vlPFC). At the first *visit*, patients underwent the SCID interview and were assessed on the items of the Hamilton Rating Scale for Depression (HAM-D) (74). Furthermore, all participants completed questionnaires and received a standardized cognitive reappraisal training and instructions about the NF training. The second *visit* entailed two baseline cognitive reappraisal runs without NF (1st run: decrease negative feelings in response to aversive pictures; 2nd run: increase positive feelings in response to pleasant pictures) as well as anatomical recordings. However, second day data is not part of the current analyses. The NF training was completed on the *third and fourth visit* with random allocation to the order of left vs. right vlPFC regulation. This was done in a double-blind manner as investigators saw the time-courses of both left and right vlPFC ROIs during the NF training, however, were blind to which ROI was used for feedback computation. Trainings were separated by at least 1 week. On each NF day, participants completed four NF runs (~7 min each), each comprised of 9 regulation blocks. Participants received intermittent numerical feedback, signaling the increase in brain activation within the target ROI. Each NF training was preceded and followed by a resting state (RS) fMRI measurement. In a *follow-up* telephone interview 4 weeks after completing the last NF training, the change of symptomatology, affect, and emotion regulation strategy use were assessed.

#### Cognitive Reappraisal Training for Emotion Regulation

A 30-min training entailed instructions and information about the NF procedure and training on the content and application of reappraisal strategies. Participants were told that the goal of the NF training was to temporarily enhance activation in a region of the brain that has been associated with the regulation of emotions. Emotional reactions to negative stimuli were discussed and participants learned to apply reappraisal strategies. In specific, participants saw negative stimuli and were instructed to use reappraisal strategies to reduce their negative affect. Suggested strategies were to think (1) the situation will change in the future, (2) the situation is not as bad as it looks. Participants could also imagine that the situation is not real or change their perspective (e.g., professional). Lastly, it was explained that a more successful cognitive reappraisal could be associated with increased activation in the target region and higher NF scores.

#### NF Task

The NF task was adapted from our previous study in patients with PTSD (71) and consisted of 18 blocks of picture presentation (12 s each; see Figure 1B). The task was either to passively view a picture and to allow spontaneous thoughts and emotions to occur (“view” condition) or to upregulate the BOLD signal in the respective target region by using a cognitive reappraisal strategy to reduce negative feelings associated with the presented picture (“reappraise” condition). The condition was indicated by a fixation cross (“x” = view; “+” = reappraise). Each picture presentation (12 s) was followed by a 4 s rest period (display of “x” or “+”) and 4 s presentation of either a numerical feedback value (1–99) after “reappraise” condition or a placeholder (“%%”) after the “view” condition, respectively. During the “reappraise” condition participants were free to try strategies related to cognitive reappraisal. They were, however, asked to stick to the same strategy during a regulation block. Prior to and following each NF run, participants indicated their emotional state using a Self-Assessment Manikin (SAM) from 1 to 9 (valence and arousal).

#### Stimuli

For the NF training, 72 pictures with negative valence were selected from the International Affective Picture System (IAPS). The IAPS contains pictures validated on valence (1–9; 1 = extremely negative) and arousal (1–9; 1 = no arousal) (75). From these 72 pictures (see Appendix A), two sets of 36 pictures were selected which were matched for overall valence and arousal (Set1: MeanValence = 2.55 ± 0.31, MeanArousal = 5.82 ± 0.53; Set2: MeanValence = 2.54 ± 0.36, MeanArousal = 5.81 ± 0.48) and the two semi-randomized versions of these sets were randomized over days. Each complete set consisted of two subsets of 18 pictures. One subset was used for Run 1 and 3 whereas the other was used for Run 2 and 4. The order of “view” and “reappraise” was switched within each subset for each repetition. Within subsets “view” and “reappraise” pictures were also matched for valence and arousal. All chosen pictures were related to one of four categories (“accident,” “assault,” “sadness,” “other”) and each “view-reappraise” cycle showed two pictures of the same category.

### Questionnaires and Neuropsychological Assessment

Symptom severity was assessed at different time points using well-established measures of depression and related features. At baseline, the patients completed the German version of the 21-item Hamilton Rating Scale for Depression (74) as well as the Becks Depression Inventory (BDI-II) (76), the Hospital Anxiety and Depression Scale (HADS) (77), the Chapman Anhedonia Scale (78), the Emotion Regulation Questionnaire (ERQ) (73), and the Heidelberg Form for Emotion Regulation Strategies (HFERST) (79). The BDI-II as well as the ERQ and HFERST were repeated on the day of the first MRI measurement if the time between baseline and first fMRI measurement was >1 week and were completed again before the first and second NF training and at follow-up. All questionnaires (excluding HAM-D) were also completed by healthy individuals. Mood as measured by the Positive and Negative Affect Schedule (PANAS) (80) was assessed at baseline, before and after each fMRI measurement and at follow-up. Furthermore, to assess meta-cognitive awareness of regulation success a short interview was completed before and after each rtfMRI NF measurement. Before each NF training (Pre-NF interview), participants were asked (1) whether they think they generally have control over their brain activity (“yes” or “no”) as well as the level of perceived control (0 = no control; 10 = a lot of control), (2) whether they think they will be able to regulate their brain signal during the NF training (“yes” or “no”) and the expected level of control (0 = not at all; 10 = very much) and (3) how successful they expect to be in using the strategy of cognitive reappraisal during the NF training (0 = not at all; 10 = very successful). Following each NF training (Post-NF interview), participants were asked (1) whether they thought they were able to regulate their brain signal during the training (“yes” or “no”) and about the level of perceived control (0–10), (2) how well the application of the cognitive reappraisal strategy worked (0 = not at all; 10 = very good) and (3) to rank how often they used the suggested (or other) strategies. Further, a selection of negative pictures viewed and reappraised during the NF training were rated on valence and arousal after each training day and the applied regulation strategies were noted. To assess cognitive performance, a verbal intelligence test (Wortschatztest – WST) (81), a working memory test measuring the capacity to store numbers (digit-span task) as well as the Digit Symbol Substitution Test were administered.

### fMRI Data Acquisition

A 3.0 T whole body scanner (Magnetom TRIO, Siemens Medical Systems, Erlangen, Germany) with a standard 20-channel head coil was used to acquire the fMRI data. For baseline cognitive reappraisal runs as well as NF runs, 230 T2^*^- weighted whole-brain functional images were recorded using echo-planar imaging (TR = 2,000 ms, TE = 28 ms, flip angle = 77°, voxel size = 3 × 3 × 3 mm, matric size = 64 × 64, ascending interleaved acquisition of 34 transverse slices, 3 mm slice thickness, 0.75 mm gap). Furthermore, high resolution T1-weighted images were acquired using a MPRAGE sequence (TE = 3.03 ms, inversion time TI = 900 ms, TR = 2,000 ms, flip angle = 9°, FOV = 256 × 256 mm2, 1 mm isotropic voxels, 0.5 mm gap, 176 sagittal slices).

### Online Real-Time fMRI Analysis

Online analysis of functional data during real-time fMRI was performed using Turbo-BrainVoyagerTM (TBV) Version 3.2 (Brain Innovation, Maastricht, NL) as elsewhere described in more detail (82). Online preprocessing included 3D motion detection and correction as well as intra session alignment for subsequent NF runs (alignment to reference volume of the first run), linear trend removal, spatial smoothing with 3 mm Gaussian smoothing kernel and temporal filtering (drift removal). Statistics were computed incrementally using a general linear model (GLM) based on the predefined stimulation protocol. The BOLD percentage signal change within the ROI was calculated using the “reappraise > view” contrast and fed back as a positive number between 1 and 99 reflecting 0–1% BOLD signal change. Feedback was computed and presented with custom scripts running under Matlab R2014a (The MathWorks Inc., Natick, MA).

#### ROI Definition

Predefined anatomical ROIs, namely the left and right vlPFC, were used for NF training. The chosen ROIs are major hubs of the cognitive reappraisal network and were based on peak coordinates provided by a meta-analysis about the neural correlates of cognitive reappraisal in healthy individuals (37). The lateral PFC has been shown to be consistently recruited during cognitive reappraisal of negative stimuli and activation in the vlPFC is altered in depression (5). For a comparable selection of left and right vlPFC, the mirrored center of the MNI coordinates of left and right vlPFC (left vlPFC: −42,22, −6; right vlPFC: 50,30, −8) (37) were selected as respective ROIs (± 46, 26, −7) and transformed to Tailarach space (± 44, 22, 3) using the “mni2tal” web application (https://bioimagesuiteweb.github.io/webapp/mni2tal.html). Based on these coordinates, ROIs (10× 10× 10 mm) were created for the left and right vlPFC using BrainVoyager QX 2.8 (Brain Innovation, Maastricht, NL). These standardized ROIs were applied using coregistration of individual anatomical scans to the Tailarach template.

#### TBV Based ROI Analysis

To test our primary hypothesis concerning a learning effect of self-control over the neuronal activity within the target ROIs we examined the feedback data recorded during NF trainings. Investigation of the learning effect is in line with the consensus paper by Ros et al. (83). To avoid confounds such as order effects, only the first day of NF training was used for this analysis. The ROI data created by TBV during online processing was exported to and analyzed in Matlab R2018. Similar to the online FB calculation, differences between regulation and view blocks were calculated for each regulation trial (4 runs each with 9 reappraise-view trials) to investigate the learning effect within runs. Learning within runs was defined as a linear increase as suggested by the “consensus” (83), computed as linear regression slope across trials for each NF run. Separate independent *t*-tests investigated differences in learning in the left and right ROI between groups and between receiving left vs. right feedback. Associations between learning success (average learning slope in vlPFC ROI) with changes in self-rating of depressive severity [BDI-II Total score (Post NF1) – BDI-II Total score (baseline)] and cognitive reappraisal [ERQ-CR Total score (Post NF1) – ERQ-CR Total score (baseline)] were calculated. Accordingly, improvement of depressive symptomatology was indicated by a negative change score whereas improvement on cognitive reappraisal use was linked to a positive change score. One-tailed testing was chosen based on the assumption of a negative relation between learning and symptoms of depression and a positive relation between learning and cognitive reappraisal use.

### Offline Data Processing and Analysis

#### Quality Assurance of MRI Data

To ensure high quality functional and structural MRI data, all data sets were examined within 48 h following recording using a standardized quality assurance pipeline developed and used by the Psychiatric Imaging Network Germany (PING; ping-rwth-aachen.de). Quality of structural data was assured by the quality parameters of the Computational Anatomy Toolbox (CAT) (84). Further, the procedure entailed the assessment of functional data within the Automated Quality Assurance toolbox (AQuA) (85). All fMRI data used for further analyses had (on average) percent signal change values below 5%. Three participants (MDD: *n* = 1) had single runs exceeding this threshold. However, the first level fMRI results of these participants did not show significant motion Artifacts upon visual inspection and were therefore included. Average PSC values did not differ between groups (HC: 2.48 ± 0.7; MDD: 2.48 ± 0.5; *t*_(74)_ = −0.02, *p* > 0.05) and indicate adequate data quality. Movement parameters did not exceed 3 mm within any NF run.

#### Preprocessing

fMRI data was preprocessed in Matlab R2018b (Mathworks Inc., Natick, MA) using the Statistical Parametric Mapping 12 toolbox (SPM12; https://www.fil.ion.ucl.ac.uk/spm/software/spm12/). To minimize T1-saturation effects, the first five volumes of each NF run were discarded for data analysis. Functional fMRI data were realigned to the first volume (6 movement parameters). Furthermore, fMRI data was co-registered to the participant's structural T1 image, smoothed with an 8 mm FWHM Gaussian kernel and normalized to the T1-weighted ICBM152 brain template of the Montreal Neurological Institute (MNI). Data from all participants was visually inspected after preprocessing to ensure adequate coregistration and normalization.

#### Whole Brain Analysis

Brain mapping analyses were performed using SPM12. On the first level, the six movement parameters were added as covariates of no interest. The main contrast of interest (reappraise > view) from the first level analysis was used in a 2 × 2 × 2 × 4 full factorial model [group (HC, MDD) × gender (female, male) × condition (Left, Right), run (NF1, NF2, NF3, NF4)]. Results were evaluated after application of a voxel-wise threshold of *p* < 0.001 and family-wise error (FWE) correction of *p*FWE < 0.05 at voxel level.

#### Generalized Psychophysiological Interaction

A generalized psychophysiological interaction (gPPI) was computed using the functional connectivity toolbox CONN (www.nitrc.org/projects/conn, RRID:SCR_009550). Signal variance that correlated with the seed region during the regulation compared to view condition (“reappraise – view”) was investigated. The bilateral vlPFC was chosen as a seed and was created based on the target regions. Second-level results were evaluated at *p* < 0.001 uncorrected voxel level and with *p* < 0.05 FDR-correction at the cluster level.

#### Statistical Analyses

Statistical analyses were performed in IBM SPSS Statistics (version 26). Independent samples *t*-tests were computed to investigate differences in baseline questionnaires (e.g., BDI-II, ERQ, neuropsychological tests, PANAS) as well as demographic measures (age, educational level) between groups. To investigate changes in symptom severity (BDI-II) and use of cognitive reappraisal strategies from baseline to follow-up measurement, two 2 × 2 × 2 [Time (NF1, NF2) × Group (MDD, controls) × Condition (L-R, R-L)] repeated measures ANOVAs were computed. Furthermore, 2 × 2 × 2 repeated measures ANOVAS [Time × Condition × Group] were calculated separately for SAM valence and arousal ratings. Lastly, to investigate the subjective experience during NF trainings, three 2 × 2 × 2 repeated measures ANOVAs (Time x Condition × Group) of metacognitive parameters of self-control (perceived intensity of general control, perceived intensity to control brain signal, perceived success to use cognitive reappraisal strategies) were computed. *Post-hoc t*-tests were performed whenever suitable. A *p*-value of < 0.05 was considered statistically significant.

## Results

### Demographic and Clinical Data

Groups did not significantly differ regarding age, (parental) education, socioeconomic status, or basic cognitive functioning such as working memory, verbal IQ, and attention (all *p* > 0.2, see Table 1) as well as in gender ratio [χ^2^_(1, 76)_ = 0.07, *p* > 0.2]. However, there were significantly more smokers in the patient group [HC: 6, MDD: 18, χ^2^_(1, 74)_ = 8.9, *p* < 0.01]. As expected, patients with depression showed elevated baseline scores on depression [BDI-II: *t*_(73)_ = −10.8, *p* < 0.001; HADS-depression: *t*_(74)_ = −9.5, *p* < 0.001] as well as HADS-anxiety scores [*t*_(74)_ = −6.9, *p* < 0.001] compared to HCs (see Table 1). On the 21-item HAM-D, patients had average scores of 16.5 (± 7.5) indicating mild to moderate depressive symptoms. Prior to the first fMRI measurement, patients showed higher negative affect [MDD: 20.0 ± 9.2, HC: 12.4 ± 4.3; *t*_(74)_ = −4.6, *p* < 0.001] and lower positive affect [MDD: 26.9 ± 6.6, HC: 32.5 ± 7.4; *t*_(74)_ = 3.5, *p* = 0.001] than healthy individuals assessed through the PANAS. Furthermore, HCs indicated to use more cognitive reappraisal strategies [*t*_(71)_ = 4.7, *p* < 0.001] and less suppression [*t*_(71)_ = −2.4, *p* < 0.05] than patients with MDD (ERQ). The HFERST subscales further supported the clinical picture with lower scores of patients on reappraisal, acceptance, problem solving (all *p* < 0.001), and social support (*p* < 0.01) and higher scores on rumination (*p* < 0.001), avoidance (*p* < 0.01), experience suppression (*p* < 0.05), and expressive suppression (*p* = 0.08) compared to HCs.

Two separate 2 × 2 × 2 repeated measures ANOVAs (Time × Group × Condition) investigated mean SAM valence and arousal ratings across NF days, conditions, and groups (Table 2). The average SAM valence ratings were similar across NF days [*F*_(1, 72)_ = 0.05, *p* > 0.2) and conditions [*F*_(1, 72)_ = 0.1, *p* > 0.2], however, significantly different between groups [*F*_(1, 72)_ = 24.9, *p* < 0.001] with higher (more positive) valence ratings for healthy individuals (6.7 ± 1.3) than patients with depression (5.4 ± 1.25). Furthermore, there was a significant group x condition interaction [*F*_(1, 72)_ = 6.4, *p* < 0.05] and *post-hoc* tests indicated that HCs showed a significant difference between conditions on the second [*t*_(35)_ = −2.1, *p* < 0.05] but not first day of NF [*t*_(35)_ = −1.1, *p* = 0.30] whereas patients with depression showed a significant difference between conditions on day 1 [*t*_(37)_ = 2.1, *p* < 0.05] but not on day 2 [*t*_(37)_ = −1.1, *p* = 0.14] of NF training. A repeated measures ANOVA of mean arousal ratings showed no significant difference between NF days [*F*_(1, 72)_ = 1.6, *p* > 0.2], between conditions [*F*_(1, 72)_ = 0.2, *p* > 0.2] or groups [*F*_(1, 72)_ = 2.5, *p* = 0.12] indicating similar arousal throughout NF trainings, across groups and conditions.

**Table 2:** Repeated-measures ANOVAs of change from baseline to follow-up (BDI-II and ERQ) and change of general perceived control across neurofeedback trainings.

**Parameter**	**Source**	***F***	***p***
Mean SAM valence ratings at neurofeedback day 1 and 2	Time	0.05	0.83
	Group	24.9	**<0.001**
	Condition	0.01	0.92
	Time × Group	0.05	0.83
	Time × Condition	3.2	0.08
	Group × Condition	6.4	**<0.05**
	Time × Group × Condition	0.22	0.64
Mean SAM arousal ratings at neurofeedback day 1 and 2	Time	1.6	0.21
	Group	2.5	0.12
	Condition	0.21	0.65
	Time × Group	1.5	0.22
	Time × Condition	0.04	0.84
	Group × Condition	0.06	0.80
	Time × Group × Condition	2.3	0.14
Perceived level of general control (scale of 1–10)	Time	7.7	**<0.01**
	Group	9.97	**<0.01**
	Condition	0.18	0.68
	Time × Group	2.4	0.13
	Time × Condition	0.98	0.33
	Group × Condition	0.17	0.68
	Time × Group × Condition	0.05	0.82
Change of symptoms of depression (BDI-II) from baseline to follow-up	Time	23.7	**<0.001**
	Group	100.3	**<0.001**
	Condition	0.12	0.73
	Time × Group	15.0	**<0.001**
	Time × Condition	1.1	0.29
	Group × Condition	0.39	0.53
	Time × Group × Condition	0.50	0.48
Change of cognitive reappraisal (ERQ-CR) from baseline to follow-up	Time	2.2	0.14
	Group	14.3	**<0.001**
	Condition	0.85	0.36
	Time × Group	5.9	0.02
	Time × Condition	0.93	0.34
	Group × Condition	0.29	0.59
	Time × Group × Condition	0.004	0.95
Change of suppression (ERQ-suppression) from baseline to follow-up	Time	0.82	0.37
	Group	3.9	**0.05**
	Condition	0.45	0.50
	Time × Group	0.0	0.99
	Time × Condition	0.58	0.45
	Group × Condition	0.22	0.64
	Time × Group × Condition	1.6	0.21

*SAM, self-assessment manikin; BDI-II, beck depression inventory-II; ERQ, emotion regulation questionnaire. Bold values highlight significance of tests*.

Different metacognitive parameters of self-control were assessed before and after each NF session (also see Appendix B). Before each NF training, participants were asked whether they think they are *generally able to control their brain activity* (yes/no) and asked for the intensity of control. On the first day, more healthy individuals than patients with MDD indicated that they generally have control over their brain activity [HC: 90%, MDD: 53%; $\chi_{\left(1,67\right)}^{2}$ = 11.2, *p* = 0.001] whereas this difference was not significant anymore at the second training [HC: 90%, MDD: 71%; $\chi_{\left(1,67\right)}^{2}$ = 3.5, *p* = 0.06]. A 2 × 2 × 2 repeated measures ANOVA [Time (NF1, NF2) × Condition (Left, Right) × Group (MDD, control); Table 2] of perceived intensity of general control (1–10) revealed a significant main effect of time [Day1: 4.99 ± 2.1, Day2: 5.61 ± 1.9; *F*_(1, 62)_ = 7.7, *p* = 0.007] as well as a significant group difference [MDD: 4.71 ± 1.7, HC: 6.03 ± 1.7; *F*_(1, 62)_ = 9.97, *p* = 0.002]. The increase of patients' positive evaluations of the ability to control one's brain activity combined with increasing perceived intensity of control indicates an increase of self-efficacy across NF days.

### NF Effects

**Figure 2:**
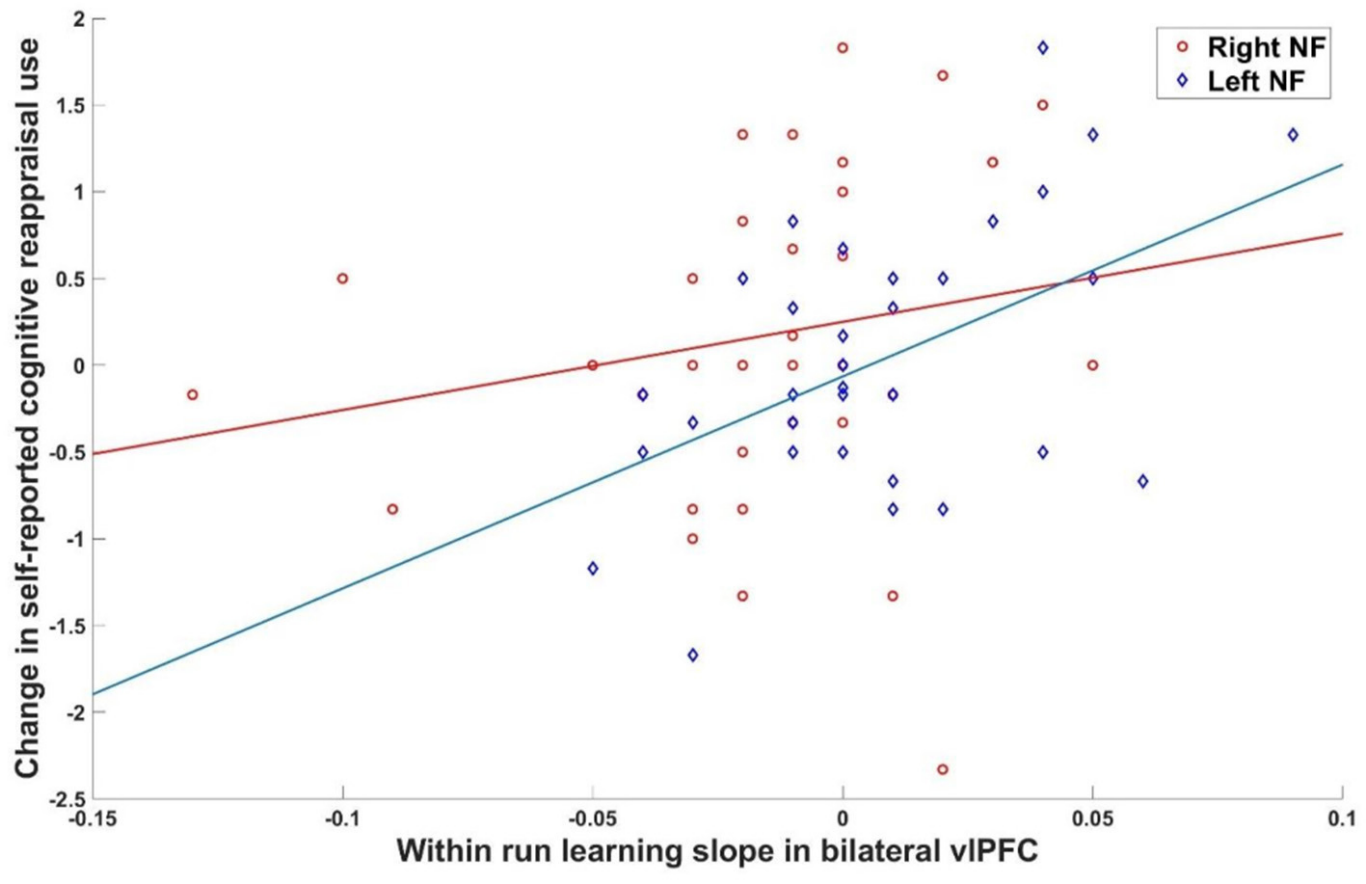
Brain behavior relationship. Association between within run learning during the first NF day and change in cognitive reappraisal use was significant for left (*r* = 0.484, *p* = 0.002) but not right (*r* = 0.170, *p* = 0.150) vlPFC feedback.

To test whether learning success was specific to the left vs. right vlPFC NF condition, ROI data of the first NF day was investigated. Within run learning slopes were steeper when receiving left as compared to right ROI feedback for both vlPFC ROIs [left vlPFC: *t*_(74)_ = 2.55, *p* = 0.01; right vlPFC: *t*_(74)_ = 3.73, *p* < 0.001]. This confirmed the primary hypothesis of a regional specific NF effect meaning that receiving feedback from the left ROI was advantageous over feedback from the right ROI. MDD and controls did not differ at either ROI [left vlPFC: *t*_(74)_ = 0.51, *p* > 0.2; right vlPFC: *t*_(74)_ = 0.96, *p* > 0.2]. Learning slopes within NF runs were significantly correlated with the change in self-reported cognitive reappraisal use from baseline to after the first NF training when receiving feedback from the left vlPFC (bilateral vlPFC ROI: *r* = 0.484, *p* = 0.002) but not from the right vlPFC (bilateral vlPFC ROI: *r* = 0.170, *p* = 0.150; Figure 2). However, there was no association between learning slopes on the first day and change of severity of symptoms of depression (left feedback: *r* = 0.09, *p* = 0.33; right feedback: *r* = 0.04, *p* = 0.41). This indicates that receiving feedback from the left as compared to the right vlPFC did not have a detectable effect on depressive symptomatology, however, left over right vlPFC feedback showed an advantage for increasing the subjective use of cognitive reappraisal strategies. Values fed back to participants during NF training are shown in Appendix C.

### Offline Analysis

#### Whole-Brain Analysis

**Figure 3:**
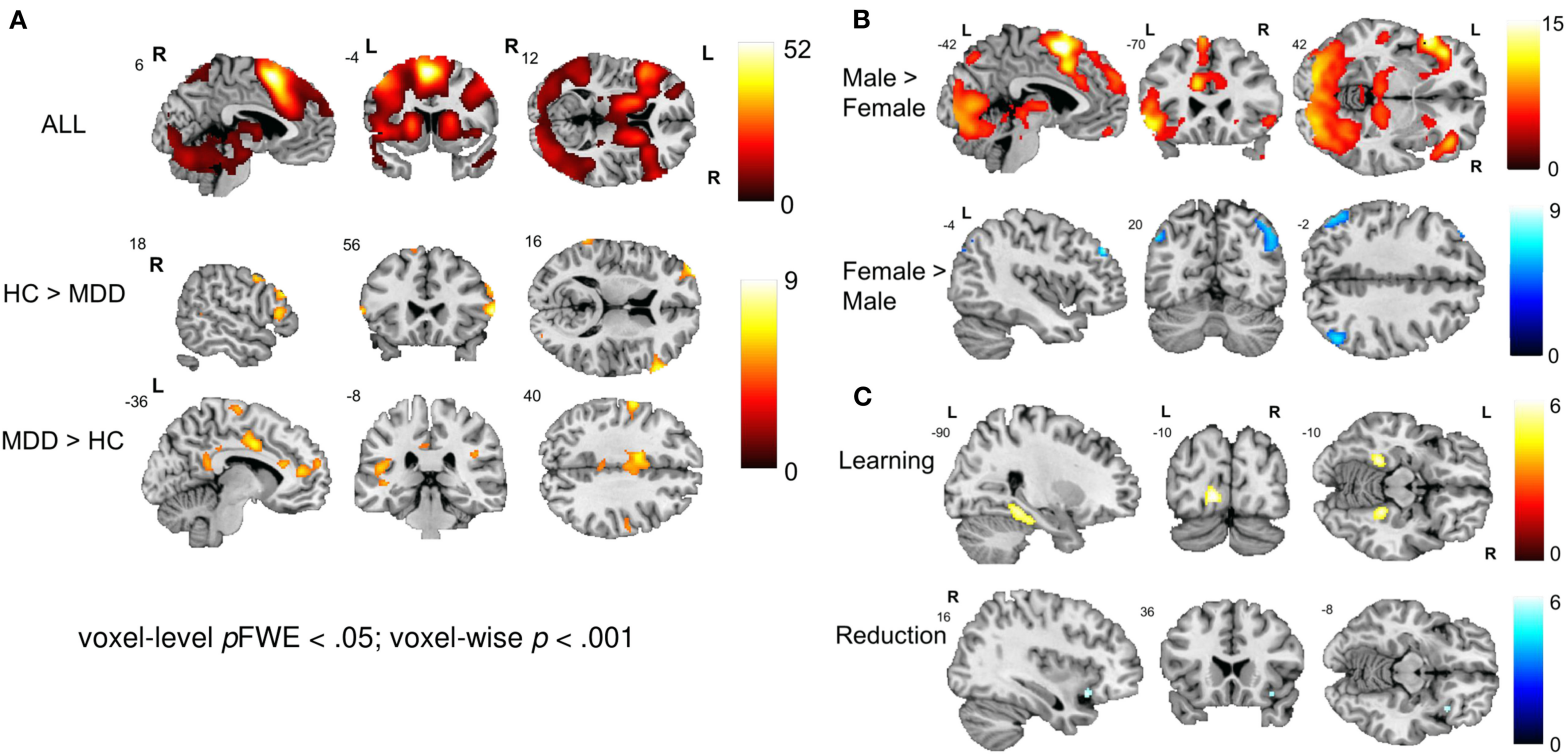
Neural correlates of emotion regulation. **(A)** The main effect of group revealed a widespread network active during NF including the bilateral prefrontal cortex, precentral gyrus, SMA, MCC, bilateral occipital and superior parietal areas, thalamus, and cerebellum. Healthy individuals showed a stronger engagement of prefrontal areas while patients showed more activation in cingulate areas. **(B)** The main effect of gender revealed engagement of an extensive network during emotion regulation in males including the bilateral prefrontal cortex, SMA, dmPFC, bilateral precentral and occipital gyrus and bilateral thalamus. Female participants showed stronger activation in the bilateral angular gyrus. **(C)** Learning and reduction across NF runs 1 to 4. Overall, participants showed an increase in the bilateral fusiform gyrus and occipital lobe as well as a decrease of activation in the right insula and the MCC.

Whole-brain activations related to NF training with cognitive reappraisal were investigated with a 2 × 2 × 2 × 4 full factorial model [Group (MDD, control) × Gender (Female, Male) × Condition (L-R, R-L) × Run (NF1, NF2, NF3, NF4)] which revealed a main effect of group showing involvement of a dorsal fronto-parietal network during NF training (Figure 3A; top; Table 3). Healthy controls exhibited more activation in the right (and to some extent left) opercular and triangular part of the inferior frontal gyrus (IFG), the right middle temporal gyrus, and left superior temporal gyrus (STG) (Figure 3A; middle; Table 3), whereas patients with MDD recruited more cingulate areas (clusters extending from anterior to posterior cingulate cortex), bilateral precuneus, bilateral pre- and postcentral gyri as well as the medial segment of the right IFG pars triangularis (see Figure 3A; bottom; Table 3). Furthermore, the main effect of condition was not significant indicating that the effect of the online TBV ROI analysis could not be detected on the whole-brain level. There was a strong main effect of gender with male participants showing more activation in the bilateral IFG, supplementary motor area (SMA), dorsomedial PFC (dmPFC), bilateral precentral gyrus, bilateral thalamus and bilateral occipital lobe (Figure 3B; top; Table 3) and female participants showing more activation in the bilateral angular gyrus (Figure 3B; bottom; Table 3). Across NF runs there was an increase in bilateral fusiform gyrus and occipital lobe as well as a decrease of activation in the right insula (Figure 3C; Table 3).

**Table 3:** Activation peaks associated with NF-guided cognitive reappraisal.

**Cluster**	**Brain region**	**MNI coordinates**			**T**	***k_***E***_***
		**x**	**y**	**z**		
**All (regulate > view)**						
1	Bilateral IFG, dorsal ACC, MCC, dmPFC, SMA, SFG, STG, angular gyrus, thalamus, striatum, occipital gyrus, pre-/postcentral gyrus, cerebellum, superior parietal lobe	−4	6	62	52.84	67,733
**Healthy controls > MDD (regulate > view)**						
1	Left MFG	−36	56	20	9.07	124
2	Right IFG	62	20	10	8.76	321
3	Right MFG	36	2	44	8.14	393
4	Right SFG	10	60	34	8.04	102
5	Left superior occipital gyrus	−24	−92	30	7.60	223
6	Right MTG	44	−42	4	6.81	82
7	Left SMA	−18	12	68	6.76	202
8	Right superior occipital gyrus	22	0.94	28	6.19	82
**MDD > Healthy controls (regulate > view)**						
1	Left anterior insula extending into frontal operculum	−34	28	10	9.02	181
2	Right pre-/postcentral gyrus	62	2	22	8.06	506
3	Left pre-/postcentral gyrus	−60	−8	42	7.45	486
4	MCC	−8	−4	42	7.05	893
5	ACC	10	38	22	6.79	441
6	Supplementary motor cortex	−6	−20	74	5.79	116
7	PCC	−12	−50	32	5.77	124
**Male > Female (regulate > view)**						
1	Bilateral IFG, pre-/postcentral gyrus, SMA, dmPFC, SFG, thalamus, striatum, occipital gyrus, left STG	−4	6	62	52.84	78,444
**Female > Male (regulate > view)**						
1	Right angular gyrus	44	−62	56	9.41	1,632
2	Left angular gyrus	−48	−70	44	6.50	386
3	ACC extending into left MFG	−12	38	−4	5.78	1,122
**Learning over time (regulate > view)**						
1	Left superior occipital gyrus	−12	−90	2	6.32	121
2	Left fusiform gyrus	−26	−44	−8	6.31	173
3	Right fusiform gyrus	24	−40	−12	5.96	249
**Reduction over time (regulate > view)**						
1	Right anterior insula	20	30	14	5.20	97

*p <0.001 voxel threshold and p <0.05 FWE-corrected at voxel level*.

#### Connectivity Analysis

**Figure 4:**
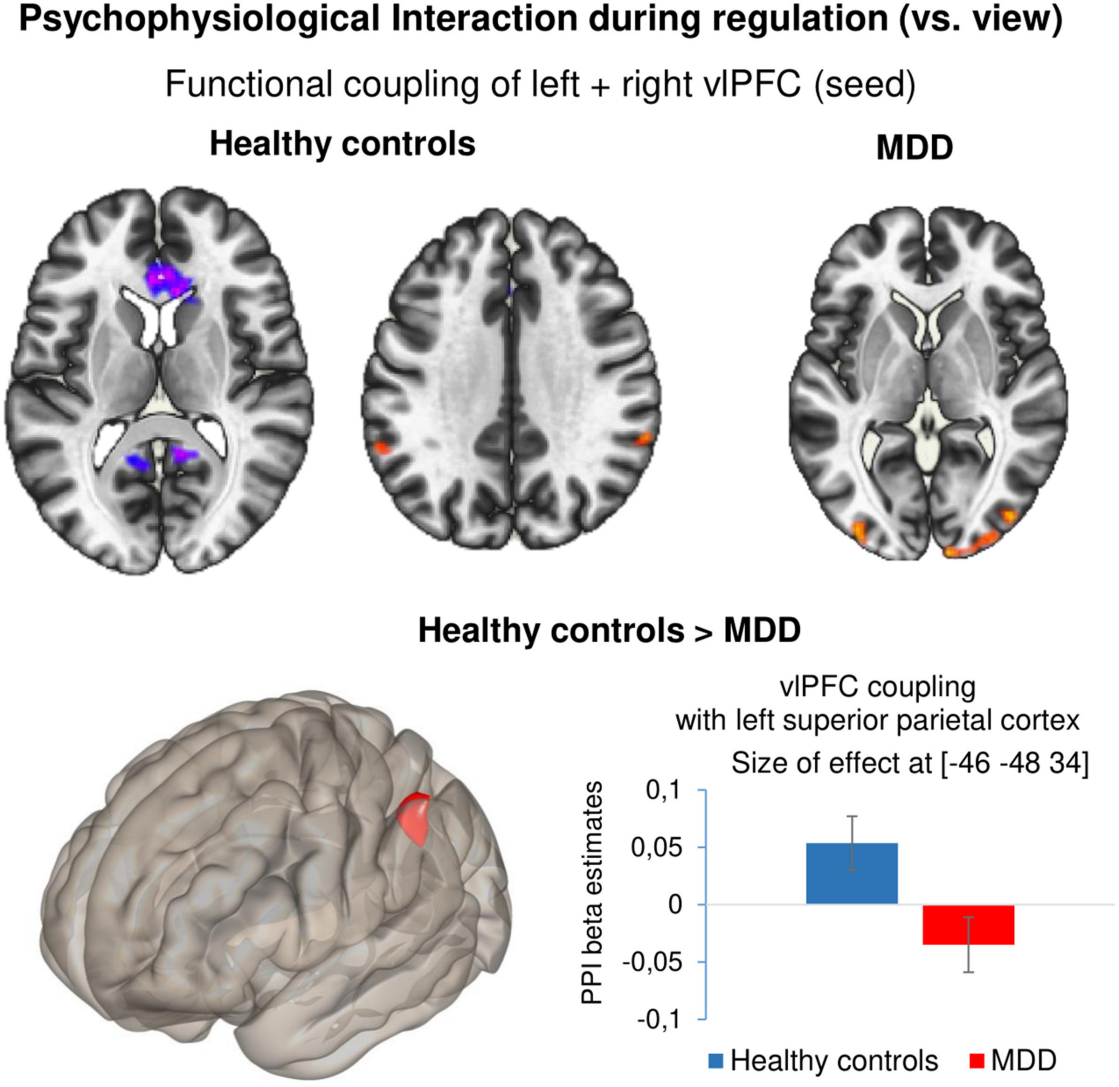
Psychophysiological interaction during regulation. Results of the generalized psychophysiological interaction analysis for the contrast ‘regulate – view’ with bilateral vlPFC as seed. Healthy controls showed increased coupling between the seed region and bilateral superior parietal cortex and negative coupling between the seed and ACC as well as PCC. Patients showed positive coupling between the seed and bilateral occipital cortex. Compared with healthy controls, patients with depression showed significantly reduced coupling between the bilateral vlPFC and left superior parietal cortex. All results are reported at *p* < 0.001 voxel threshold and *p* < 0.05 FDR-corrected at cluster level. Bar plots indicate size of effect (beta values) at the voxel showing the maximum coupling effect.

Task-dependent changes in functional connectivity of the bilateral vlPFC during neurofeedback was studied with a generalized Psychophysiological Interaction analysis in the contrast reappraise vs. view. In healthy controls, coupling increased with the left (*x* = −58, *y* = −52, *z* = 46, *T* = 4.64) and right superior parietal cortex (*x* = 60, *y* = −42, *z* = 32, *T* = 4.68) and decreased with the ACC (*x* = 12, *y* = 18, *z* = 0, *T* = −6.69), precuneus/PCC (*x* = 4, *y* = −38, *z* = 8, *T* = −5.27), right superior frontal gyrus (*x* = 24, *y* = 30, *z* = 44, *T* = −4.81). In the patients, neurofeedback enhanced coupling with the left (*x* = 10, *y* = −102, *z* = 2, *T* = 5.14) and right inferior occipital gyrus (*x* = −34, *y* = −94, *z* = 0, *T* = 5.03; *x* = 44, *y* = −82, *z* = 10, *T* = 4.56). Direct group comparison revealed significantly lower functional connectivity during NF in the left superior parietal cortex in patients compared with healthy controls (*x* = −46, *y* = −48, *z* = 34, *T* = −6.69) (Figure 4). In a *post-hoc* analysis, baseline BDI scores were negative linear predictors for the connectivity measure at this location [*T*_(72)_ = −2.99, *p* = 0.004].

### Follow-Up Assessments

Four weeks after the last NF training, all participants were contacted to fill out questionnaires on their current depressive symptomatology, their state of affect, their use of emotion regulation strategies and experience with the NF training as well as applications of learned strategies in everyday life. Not all participants could be recontacted for follow-up assessments (MDD: 32, HC: 35) and the following analyses are based on the available subset of data. Please see Table 2 for detailed results.

#### Symptom Change

**Figure 5:**
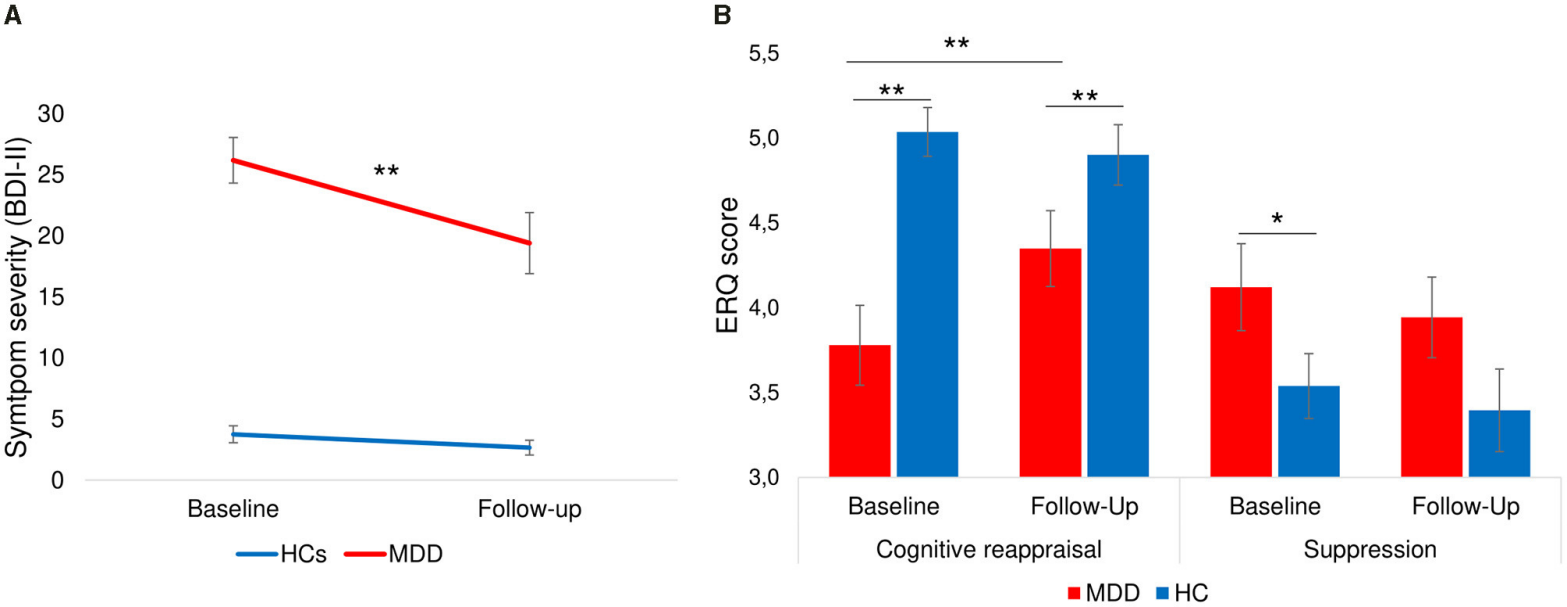
Change of severity of depression and emotion regulation ability from baseline to follow-up. **(A)** Symptom severity measured by BDI-II showing a significant decrease of symptom scores from baseline to follow-up only in patients with depression [9.2 ± 11.8; *t*_(28)_ = 4.2, *p* < 0.001]. **(B)** Patients showed a significant increase of cognitive reappraisal and a stable level of suppression strategies. Healthy individuals had stable levels of cognitive reappraisal and suppression. Error bars indicate standard errors. **p < 0.001, *p < 0.05.

A 2 × 2 × 2 [Time (Baseline, Follow-up) × Group (HC, MDD) × Condition (L-R, R-L)] repeated measures ANOVA of depressive symptom severity (BDI-II) showed a significant main effect of time [*F*_(1, 60)_ = 23.7, *p* < 0.001], a significant effect of group [*F*_(1, 60)_ = 100.3, *p* < 0.001] whereas the main effect of condition did not reach significance [*F*_(1, 60)_ =.12, *p* = 0.73]. Furthermore, the time^*^group interaction was significant [*F*_(1, 60)_ = 15.0, *p* < 0.001] while the time^*^condition interaction did not show significance [*F*_(1, 60)_ = 1.1, *p* = 0.29]. *Post-hoc* paired *t*-tests showed that the reduction of symptom severity from baseline to follow-up was significant for patients [9.2 ± 11.8; *t*_(28)_ = 4.2, *p* < 0.001, *d* = 0.77; Figure 5A] but not for HCs [1.0 ± 3.3; *t*_(34)_ = 1.9, *p* = 0.07, *d* = 0.24]. Previous studies have shown that the minimal clinically important difference (MCID) of BDI-II scores should be a change of at least 5 points (86) or 17.5% (87) depending on the baseline severity of depression. The mean change of 9.2 points (27.7% reduction) in our sample indicates that patients on average showed a meaningful reduction of BDI-II scores from baseline to follow-up. Furthermore, investigation of MCID on an individual level indicated a clinically meaningful change (change ≥5 or 17.5%) in 55% of patients who completed the follow-up interview.

#### Change of Emotion Regulation (ERQ)

A 2 × 2 × 2 [Time (Baseline, Follow-up) ×Group (HC, MDD) × Condition (L-R, R-L)] repeated measures ANOVA of cognitive reappraisal use at baseline and follow-up showed non-significant main effects of time [*F*_(1, 59)_ = 2.2, *p* = 0.14] and condition [*F*_(1, 59)_ = 0.85, *p* = 0.36], but a significant main effect of group [*F*_(1, 59)_ = 14.3, *p* < 0.001] as well as significant time^*^group interaction [*F*_(1, 59)_ = 5.9, *p* = 0.02]. *Post-hoc* tests (see Figure 5B) indicated that patients had a significant increase in cognitive reappraisal use [*t*_(30)_ = −2.5, *p* = 0.02, *d* = 0.43] from baseline to follow-up, whereas healthy individuals showed a stable use of cognitive reappraisal [*t*_(31)_ = 0.80, *p* > 0.2]. The difference between groups remained significant at follow-up [*t*_(64)_ = 2.1, *p* < 0.05]. A similar repeated measures ANOVA of self-reported suppression revealed only a marginally significant main effect of group [*F*_(1, 59)_ = 3.9, *p* = 0.05]. *Post-hoc* tests indicated that the difference between groups for suppression that was significant at baseline [*t*_(71)_ = 2.1, *p* = 0.02] was not significant at follow-up [*t*_(64)_ = −1.4, *p* > 0.1], indicating that use of suppression in patients equalized with that of healthy individuals. Interestingly, male participants used more suppression compared to female participants at baseline [*t*_(71)_ = −2.6, *p* < 0.01] and follow-up [*t*_(64)_ = −2.3, *p* = 0.02] whereas there were no gender differences for cognitive reappraisal use at baseline [*t*_(71)_ = 0.72, *p* > 0.2] or follow-up [*t*_(64)_ = 0.14, *p* > 0.2].

#### Subjective Experience

At follow-up, the application of learned strategies in everyday life was assessed. Seventy-five percentage of patients with MDD and 57% of healthy individuals indicated that they had used the strategies over the past month. Of these, all patients and 89% of controls experienced the applied strategies as helpful. Patients reported that reappraisal strategies made them feel less frustrated, more relaxed, more optimistic, more aware of the situation, and led to improved mood which indicates that generalization of the training to negative situations in everyday life was high. Furthermore, 89% of controls and 91% of patients were willing to repeat such a NF training which indicates high acceptance of the NF training.

## Discussion

In this double-blind cross-over rtfMRI study we tested the feasibility and clinical efficacy of NF-supported cognitive reappraisal training in patients with depression and a matched group of healthy individuals. In specific, we investigated whether left- or right hemispheric ventrolateral prefrontal cortex (vlPFC) NF would be a neurologically and clinically more suitable region for NF during cognitive reappraisal. During 2 days of NF training, participants trained to regulate their brain signal in the vlPFC using reappraisal strategies in response to negative pictures and guided by intermittent NF (“reappraise > view”). This paradigm has already been applied successfully in healthy individuals (70) and patients with post-traumatic stress disorder (PTSD) (71). Overall, the cognitive reappraisal evoked predicted responses within the emotion regulation network such as activations in prefrontal, motor, and subcortical areas. Furthermore, our ROI analysis revealed that NF of left compared to right vlPFC activity specifically enhanced bilateral prefrontal activation during reappraisal in patients with depression as well as in age and gender matched healthy individuals. Such laterality effect did not survive the correction for multiple testing in the whole-brain analysis. First, it has to be taken into account that the size of lateralization effects is usually limited, e.g., about 30% lower responses at the non-dominant hemisphere to speech stimuli (88). Secondly, recent data show a relevant contribution of the right vlPFC [e.g., (50)]. However, a larger learning effect in response to left vs. right vlPFC feedback in the ROI analysis suggests a regional specificity of our rt-fMRI-based NF paradigm and may further support a central causal role of this region for cognitive reappraisal (41, 52, 89).

NF learning on the first day of training was related to improvements in self-rated reappraisal use only when receiving NF from the left rather than right vlPFC but was not associated with change of depressive symptomatology. Nevertheless, 55% of patients showed a clinically meaningful change in depression scores (BDI-II) from baseline to follow-up and 75% of patients reported that they had successfully applied the learned cognitive strategy in everyday life. Our study supports a positive effect of rt-fMRI NF for the enhancement of emotion regulation for patients with depression (90). The combination of specific NF effects, reappraisal skill learning, reduction of depressive symptomatology and a high acceptance of training yield the (especially left) vlPFC a promising target for future NF-guided rt-fMRI studies.

Behaviorally, patients with depression can achieve downregulation of negative emotions by applying reappraisal strategies within clear laboratory boundaries. However, Zilverstand et al. (5) have shown that patients with depression may – despite similar behavioral regulation success – display dysfunctions in a cognitive control network for negative emotions of which the vlPFC offers a promising NF target. Our findings from the first NF day suggest a beneficial effect of receiving left compared to right vlPFC feedback reflected by enhanced bilateral vlPFC activation and a significant increase in reappraisal strategy use specific for left vlPFC feedback. The vlPFC has repeatedly been found to support the selection of appropriate reappraisals (37, 38) and left vlPFC activity during reappraisal differentiates healthy individuals from patients with depression (5, 40). Furthermore, more evidence for a superior role of the left compared to right vlPFC for cognitive reappraisal has been provided by previous studies (52, 59).

Further investigation on the whole-brain level showed that healthy individuals and patients with depression recruited different regions of the emotion regulation network during NF-supported reappraisal training. The effect of interest showed extensive activation in the bilateral vlPFC, dlPFC, dACC, (pre-) SMA, dmPFC, SFG, MFG, STG, angular gyrus, thalamus, striatum, occipital gyrus, pre- and postcentral gyrus, cerebellum and superior parietal lobe during cognitive reappraisal. This is consistent with the emotion regulation network areas commonly recruited during reappraisal (36–38). Furthermore, in the group comparison, healthy individuals displayed more recruitment of cortical areas including the right IFG, left SMA, bilateral middle frontal gyrus, right superior frontal gyrus, right middle temporal gyrus and the bilateral superior occipital gyrus. Deficient recruitment of prefrontal areas (vlPFC, dlPFC) (5) and the STG (40) as seen in patients with depression is in line with dysfunctional attentional and inhibitory capacities. Interestingly, we did not find any effects in the amygdala and therefore could neither replicate the attenuation of amygdala response during reappraisal training found in previous studies (70, 71) nor the group difference in terms of increased amygdala response during reappraisal (5). Accordingly, findings in the current cohort may support a direct pathway hypothesis postulating that reappraisal primarily impacts cortical systems involved in cognitive appraisals and emotional evaluation and only has a minimal impact on subcortical systems (41). Whether distinct regulation pathways during NF-guided cognitive reappraisal reflect specific diagnoses or symptom clusters, must be determined in further investigations. Across NF runs, the analysis revealed a significant increase within the bilateral fusiform gyrus as well as occipital lobe which may indicate increased processing of visual stimuli over time. Additionally, we observed a decrease of a cluster extending from the ACC to the right anterior insula which may be related to either a habituation to emotional stimuli and accordingly reduced salience of the displayed negative pictures or a reduction of salience due to more effective reappraisal application.

Patients with depression, showed more activation within midline areas (ACC, MCC, PCC, precuneus), pre- and postcentral gyrus, Supplementary motor cortex as well as the left anterior insula compared with control participants. Increased activation within the left anterior insula and the precentral gyrus in patients may be related to increased emotional experience (40). This pattern may suggest amplified physiological and motor responses resulting from increased processing of perceptual information of negative stimuli. In particular, the anterior insula is involved in the integration of external environment and interoceptive physiological signals received from the posterior insula (91, 92). Furthermore, increased activation of the precentral gyrus may stem from more intense emotion experience [e.g., (93)]. Additionally, the ACC has been related to the appraisal and expression of negative emotions (94), upregulation of this area by fMRI NF has been shown to modulate emotion perception (95) and has been related to increased rumination (96). On the other hand, higher activation levels of the MCC, PCC, and SMA in patients compared to healthy individuals may indicate a compensatory mechanism (97, 98).

During emotion regulation, the (anterior) MCC has been suggested to play an integratory role mediating between emotion appraisal in subcortical and initiation of reappraisal in prefrontal regions and relaying the need to regulate from the vlPFC to the dlPFC (37). As a hub of the default-mode network (DMN), the PCC is involved in inward attention and self-reflective thinking (99, 100). The PCC is critically involved in reappraisal (37, 38) and higher involvement of this area during NF in patients with depression was unexpected. However, it is possible that increased activation of MCC and PCC reflect a compensatory mechanism that counteracts deficiencies of the fronto-parietal attention network. For instance, PCC activation has been related to effortful cognitive control (101). In a similar vein, we found compensatory engagement of prefrontal regions during self-control of brain activation in a previous neurofeedback study in PTSD (13). Alternatively, it is conceivable that patients used more self- compared to situation-focused reappraisal strategies and therefore evoked more activation in self-monitoring brain state [e.g., (102, 103)]. The increased activation in the SMA, a region involved in the execution of regulation (37), may signal increased effort of reappraisal in patients. From a network perspective, these results suggest dysfunction in the cortical cognitive control network responsible for negative emotions with exaggerated brain responses related to increased emotional experience. Furthermore, Morawetz et al. (104) identified four large scale networks involved in emotion processing and regulation. One of the suggested networks comprised of the bilateral postcentral gyrus, left insula and PCC as well as periaqueductal gray and left superior parietal lobe shows large overlap with the pattern of activation seen in patients with depression. Further, the authors propose that this network may serve as an emotion regulation hub which integrates information from a ventral and dorsal prefronto-parietal regulation and a subcortical emotion generation network.

The task-based connectivity analysis during NF revealed enhanced coupling in healthy individuals between the seed (bilateral vlPFC) and the bilateral superior parietal cortex as well as an inhibitory effect on ACC, PCC, precuneus and the right superior frontal gyrus. Patients showed an increased coupling with the bilateral occipital lobe which may indicate increased attention toward negative emotional stimuli (105). Importantly, in a group comparison, patients with depression showed lower functional connectivity of the bilateral vlPFC and the left superior parietal cortex. Reduced connectivity between these regions has been associated with less recruitment of the regulatory fronto-parietal network involved in reappraisal and subsequent inefficient cognitive transfer of information from frontal to parietal areas (40, 106). Furthermore, weaker connectivity within a fronto-parietal attention network in depression has been related to poorer goal-directed attention (107) and seems to be a general impairment of attention that has implications for different sensory domains (108, 109). Lastly, baseline depression severity (BDI-II) was an inverse predictor of functional connectivity at this location indicating that individuals suffering from more severe symptoms showed particularly prominent decoupling in this network.

Interestingly, we observed strong gender differences in brain activation related to reappraisal. Investigation of gender differences has frequently yielded mixed findings in the literature (43). Our whole brain analysis showed that whereas females displayed enhanced activation in the bilateral superior parietal cortex, males showed a widespread pattern of activation within bilateral IFG, SMA, dmPFC, bilateral precentral gyrus, thalamus, and occipital lobe during reappraisal. On the behavioral level, these differences are underpinned by more everyday life suppression strategy use in males in both groups – patients with depression and healthy individuals. Interestingly, several studies show that males and females display different neural responses during reappraisal tasks despite comparable decreases of negative affect (44, 110). However, contrary to our findings, McRae et al. (110) showed less increase in prefrontal regions in males compared to females during a reappraisal task. Furthermore, increased effective fronto-limbic connectivity in males compared to females during negative emotion processing indicates that men show more evaluative rather than affective processing of negative emotional stimuli (111). Whereas, males have also been shown to use more automatic, non-conscious emotion regulation, females are more likely to focus on and analyze their negative emotions (112). Furthermore, disturbed perception of appraisals has been observed in female patients with depression (113). Our findings suggest that males and females recruit different neural pathways during reappraisal. Alternatively, Whittle et al. (44) highlight females are more neurally reactive to disgust, anger and fear related negative emotional stimuli. This could imply that the observed gender differences may reflect differences in our higher-level baseline (viewing condition) which may have differentially affected the differences between reappraisal and viewing blocks in males and females. Even though the origin of the observed differences in gender-specific neural responses remains speculative at this point, our results underscore the importance of the investigation of gender effects in the context of emotion regulation.

In the current NF study, patients with depression reported a positive effect of reappraisal use such as less frustration, more relaxed handling of emotions, more awareness of negative situations as well as improved mood during the 4-week period following NF training. Our study extends the findings of our previous studies using NF supported reappraisal training for downregulation of negative emotions in healthy individuals (70) and patients with PTSD (71) and implies a possible pathway for neural enhancement-based treatment strategies in patients with depression even after short training periods. Cognitive reappraisal strategies during the 4 weeks following NF training were utilized by 75% of both patients with depression and PTSD (71). Furthermore, we could show a similar clinically meaningful effect size of depressive symptomatology change from baseline to follow-up (*d* = 0.77) as in patients with PTSD (*d* = 0.64) (71) suggesting a potential of the NF training across diagnostic categories. As NF learning was accompanied by an overall increase in perceived intensity of control over brain activation, the improvement of depressive symptomatology may possibly be related to an increase of self-efficacy beliefs associated with a non-specific reward experience of self-regulation (67). Mastery experiences build strong self-efficacy beliefs (114) and may help to overcome learned helplessness which is a common experience of patients with depression (115). As MacDuffie et al. (8) have stated, a lack of experience of the beneficial effects of newly learned cognitive skills for patients with depression may complicate their transfer to complex day to day situations. The direct objective visual feedback on neural effects of strategy use during NF may have enhanced the credibility of strategy use and motivated application in real-life.

Importantly, the observed clinical improvements in the current study, cannot be directly attributed to specific NF effects due to the nature of the cross-over design; in particular, we cannot differentiate effects of left and right vlPFC NF, the reappraisal training, placebo [see (67)] as well as time passed (116). It has to be noted that even in the direct comparison of left vs. right feedback ROI data, only moderate *t*-values were achieved (2.55 and 3.69), suggesting a small to moderate effect size only. In consequence the finding did not emerge in the brain mapping analysis with correction for multiple testing across voxels. On the other hand, considering that both the left and right vlPFC are involved in the process of reappraisal [e.g., (37)] finding a difference between regulation conditions is rather impressive. However, to allow a systematic investigation of reappraisal enhancing effects of vlPFC NF, future studies should use a between-subjects design comparing this target region with a control feedback condition. For example, the implementation of a different control condition such as a region that is not involved in reappraisal but of which participants can achieve similar control may be advantageous [e.g., (64)]. Furthermore, to disentangle effects of NF training and use of emotion regulation strategies as such, a third control group without neurofeedback is essential. Another important aspect concerns the observation that clinical symptoms continue to improve for weeks after treatment (117). To understand the trajectory of NF effects on cognitive strategy use, more frequent follow-up assessments and longer follow-up intervals should be implemented. Furthermore, objective evaluation of treatment success at follow-up such as a reappraisal transfer run may avoid a possible bias of retrospective self-report questionnaires and interview.

## Conclusion

Our findings support a central role of the left vlPFC for the process of cognitive reappraisal. Left compared to right vlPFC NF was associated with increased bilateral frontal self-regulation and improved emotion regulation. Further, we showed differences in the specific recruitment of emotion regulation areas between patients with depression and healthy individuals as well as between females and males during cognitive reappraisal. Inefficient execution of emotion regulation in patients with depression was further supported by weaker task-based connectivity in the fronto-parietal attention network that was associated with symptom severity at baseline. Our findings suggest a good tolerability of our rtfMRI-NF-guided cognitive reappraisal training and potential for clinical use in patients with depression. Randomized clinical trials with longer follow-up intervals and additional control groups are needed to validate this potential.

## Data Availability Statement

Data used in this study is not available due to legal restrictions. Requests to access the datasets should be directed to kmathiak@ukaachen.de.

## Ethics Statement

The studies involving human participants were reviewed and approved by local Ethics Committee of the RWTH Aachen. The patients/participants provided their written informed consent to participate in this study.

## Author Contributions

MKe, JZ, MKl, MZ, and KM contributed to conception and design of the study. MKe, RM, and JI were involved in the investigation. MKe and KM performed the statistical analysis. MKe wrote the manuscript. All authors corrected the manuscript and approved the submitted version.

## Conflict of Interest

The authors declare that the research was conducted in the absence of any commercial or financial relationships that could be construed as a potential conflict of interest.

## Publisher's Note

All claims expressed in this article are solely those of the authors and do not necessarily represent those of their affiliated organizations, or those of the publisher, the editors and the reviewers. Any product that may be evaluated in this article, or claim that may be made by its manufacturer, is not guaranteed or endorsed by the publisher.
