# Supplementary material for: fMRI Neurofeedback-Enhanced Cognitive Reappraisal Training in Depression: A Double-Blind Comparison of Left and Right vlPFC Regulation
Source: Front Psychiatry. 2021 Aug 23;12:715898. doi: 10.3389/fpsyt.2021.715898 (PMC8419460; doi:10.3389/fpsyt.2021.715898)
Supplement: Supplementary file 1 [file Data_Sheet_1.docx]

Supplementary Material

**Appendix A. IAPS numbers**

SET 1: 2053, 2141, 2375, 2691, 2799, 2900, 2981, 3103, 3160, 3220, 3230, 3550, 6021, 6210, 6242, 6250, 6300, 6315, 6415, 6540, 6571, 8485, 9042, 9050, 9120, 9250, 9419, 9428, 9435, 9600, 9611, 9623, 9630, 9900, 9901, 9911

SET 2: 1525, 2345, 5973, 6263, 6312, 6370, 6562, 6570, 9140, 9184, 9254, 9332, 9400, 9414, 9415, 9423, 9425, 9427, 9430, 9500, 9520, 9530, 9560, 9610, 9800, 9810, 9902, 9904, 9908, 9909, 9910, 9920, 9922, 9925, 9927, 9941

**Appendix B. *Additional information on subjective experience***

Participants indicated before and after each NF training whether they think they *will be (before)* and *were* *able to (after) regulate their brain activity during the NF training* and indicate their *perceived level of control (1-10)*. Most participants expected to be able to regulate their brain signal during NF training (HC: Day1 (Pre: 100%, Post: 89%), Day2 (Pre: 100%, Post: 92%); MDD: Day1 (Pre: 86%, Post: 83%), Day2 (Pre: 94%, Post: 86%). A 2x2x2x2 repeated measures ANOVA (Time[NF1, NF2] x PrePost[PreNF, PostNF] x Condition[Left, Right] x Group[MDD, control]) showed a significant increase of perceived control from Day1 to Day2 (main effect time: *F*(1,63) = 5.37, *p* < .05; NF1: 5.37 ± 1.6, NF2: 5.67 ± 1.6) and a significant decrease from before to after each NF training (main effect prepost: *F*(1,63) = 4.8, *p* < .05; Pre: 5.7 ± 1.6, Post: 5.4 ± 1.8) whereas there was no significant effect of group (*F*(1,63) = 2.95, *p* = .09) or condition (*F*(1,63) = .71, *p* = .4). These findings show that perceived control to regulate the brain activation increased throughout the training despite decreases of ratings observed from before to after each training.

Furthermore, participants were asked before and after the NF to rate their (expected) success to use the trained cognitive reappraisal strategies during the NF training. A similar 2x2x2x2 repeated measures ANOVA as for perceived control was used and revealed a significant effect of group (*F*(1,63) = 4.49, *p* < .05) with higher ratings by healthy individuals (HC: 6.25, MDD: 5.53) and a significant main effect of change from before to after NF (*F*(1,63) = 4.3, *p* < .05) which showed a decrease of ratings (NF1: 6.07, NF2: 5.71). Furthermore, there was a significant interaction of group and change over sessions (*F*(1,63) = 4.2) and post-hoc tests showed that this interaction was due to a non-significant increase of ratings in patients with depression (Day 1: 5.4; Day 2: 5.6) and a non-significant decrease of ratings for healthy individuals (Day 1: 6.4; Day 2: 6.1).

**Appendix C. *Mean feedback values presented to participants during NF training (Mean ± SD).***

|  | **Left vlPFC feedback** | | | | **Right vlFPC feedback** | | | | **Left** | **Right** |
| --- | --- | --- | --- | --- | --- | --- | --- | --- | --- | --- |
|  | **Run1** | **Run2** | **Run3** | **Run4** | **Run1** | **Run2** | **Run3** | **Run4** | **Run1-4** | **Run1-4** |
| **HC** | 35,7 (±15.8) | 36,3 (±17.2) | 34,2 (±16.5) | 37,5 (±17.9) | 33,8 (±17.7) | 33,8 (±13.5) | 33,9 (±13.9) | 33,7 (±15.7) | 35,9 (±13.6) | 33,8 (±11.0) |
| **MDD** | 34,7 (±18.4) | 32,8 (±15.4) | 31,1 (±18.9) | 35,6 (±21.1) | 34,7 (±18.3) | 33,4 (±19.3) | 34,3 (±15.9) | 35,1 (±18.5) | 33,6 (±15.5) | 34,4 (±15.6) |
| **ALL** | 35,2 (±17.1) | 34,5 (±16.3) | 32,6 (±17.7) | 36,5 (±19.5) | 34,3 (±17.9) | 33,6 (±16.6) | 34,1 (±14.9) | 34,4 (±17.1) | 34,7 (±14.6) | 34,1 (±13.5) |
